# Effect of Intrapartum Antibiotics Prophylaxis on the Bifidobacterial Establishment within the Neonatal Gut

**DOI:** 10.3390/microorganisms9091867

**Published:** 2021-09-02

**Authors:** Silvia Saturio, Marta Suárez, Leonardo Mancabelli, Nuria Fernández, Laura Mantecón, Clara G. de los Reyes-Gavilán, Marco Ventura, Miguel Gueimonde, Silvia Arboleya, Gonzalo Solís

**Affiliations:** 1 Department of Microbiology and Biochemistry of Dairy Products, Instituto de Productos Lácteos de Asturias (IPLA-CSIC), 33300 Villaviciosa, Spain; silvia.saturio@ipla.csic.es (S.S.); greyes_gavilan@ipla.csic.es (C.G.d.l.R.-G.); mgueimonde@ipla.csic.es (M.G.); 2 Institute of Health Research of the Principality of Asturias (ISPA), 33011 Oviedo, Spain; msr1070@hotmail.com (M.S.); nuriajmhd@gmail.com (N.F.); laura_mantecon@hotmail.com (L.M.); gsolis@telefonica.net (G.S.); 3 Pediatrics Service, Central University Hospital of Asturias (HUCA-SESPA), 33011 Oviedo, Spain; 4 Laboratory of Probiogenomics, Department of Chemistry, Life Sciences and Environmental Sustainability, University of Parma, 43121 Parma, Italy; leonardo.mancabelli@genprobio.com (L.M.); marco.ventura@unipr.it (M.V.); 5 Pediatrics Service, University Hospital of Cabueñes, 33394 Gijon, Spain

**Keywords:** *Bifidobacterium*, ITS, q-PCR, gut microbiota, antibiotics, IAP, GBS, C-section

## Abstract

Antibiotics are important disruptors of the intestinal microbiota establishment, linked to immune and metabolic alterations. The intrapartum antibiotics prophylaxis (IAP) is a common clinical practice that is present in more than 30% of labours, and is known to negatively affect the gut microbiota composition. However, little is known about how it affects to *Bifidobacterium* (sub)species level, which is one of the most important intestinal microbial genera early in life. This study presents qualitative and quantitative analyses of the bifidobacterial (sub)species populations in faecal samples, collected at 2, 10, 30 and 90 days of life, from 43 healthy full-term babies, sixteen of them delivered after IAP use. This study uses both 16S rRNA–23S rRNA internal transcribed spacer (ITS) region sequencing and q-PCR techniques for the analyses of the relative proportions and absolute levels, respectively, of the bifidobacterial populations. Our results show that the bifidobacterial populations establishment is affected by the IAP at both quantitative and qualitative levels. This practice can promote higher bifidobacterial diversity and several changes at a compositional level. This study underlines specific targets for developing gut microbiota-based products for favouring a proper bifidobacterial microbiota development when IAP is required.

## 1. Introduction

Antibiotics are lifesaving drugs, due to their powerful ability to battle infections. Since the discovery of penicillin in 1928, numerous antibiotics have been described [1]. Among them, beta-lactams are the most-commonly administered compounds and make up 65% of the antibiotic market [2]. Antibiotics are also extensively used for prophylactic purposes, and they are by far the most common prescription drugs given in the perinatal and neonatal environment, being present in more than 30% of labours [3,4,5]. In this context, the administration of intrapartum antibiotic prophylaxis (IAP) is commonly used for the prevention of early-onset Group-B-streptococci (GBS) infection, in pre-labour rupture of membranes or C-section deliveries to avoid surgical infections [3,6]. However, the use of antibiotics also presents disadvantages, such as promoting antimicrobial resistance, adverse drug events or alterations of the gut microbiota [7,8,9].

The gut microbiota is an ever-changing and complex microbial ecosystem harboured by the gastrointestinal tract (GIT). The microbiota is a key factor in a range of biological processes in the host [10,11,12,13]. After birth, the GIT is rapidly colonised by a wide diversity of microorganisms. Accumulating evidence shows that a correct establishment of this microbiota in the early days of life plays an important role in the reduction of the later development of chronic diseases, such as obesity, allergies, infections, inflammatory or brain disorders, and is an overall determinant for the health of the individual [14,15,16]. Thereby, the period of the gut microbiota colonisation and establishment constitutes a critical window-of-opportunity for its modulation towards a healthy status [17,18,19]. The gut microbiota establishment begins with the settlement of facultative anaerobes and aerotolerant microorganisms, such as enterobacteria or lactobacilli, which pave the way by reducing the oxygen in the gut to the colonisation by other strict anaerobic bacteria, such as bifidobacteria or bacteroides [20,21]. Several factors have an impact in shaping the gut microbiota colonisation process [6,14,19,22,23]. Among them, antibiotics are one of the most pivotal factors promoting alterations on the intestinal microbiota establishment at the beginning of life, linked to immune and metabolic alterations [24,25,26].

Animal studies have shown that when the early-life gut microbiota is altered by antibiotics, enduring physiological effects are observed, even though there is a later microbiota restoration [27,28,29,30,31]. On the other hand, different epidemiological studies have demonstrated the association between early exposure to antibiotics and different diseases, such as allergy [32,33], asthma [31,34], celiac disease [35,36], overweight [37,38,39,40] or inflammatory bowel disease [41]. In this regard, it is proven that early-life antibiotic treatment disrupts the proper and natural development of gut microbiota with potential negative influence on later health [42,43,44]. During the last years, several studies have seen the advent reporting an impact on the gut microbiota after IAP treatment. Lower relative proportions of Actinobacteria and Bacteroidetes and increased of Proteobacteria and Firmicutes were observed during the first weeks of life, with *Bifidobacterium* being one of the most affected genera [45,46,47,48]. Nogacka et al., by using 16S rRNA gene profiling of faeces, observed reduced proportions of *Bifidobacteriaceae* family in full-term babies during the first days of life after IAP treatment [45], which is in good agreement with the results obtained by Corvaglia et al. by using q-PCR for *Bifidobacterium* genus [47]. Moreover, Mazzola et al. [46] also reported lower levels of *Bifidobacterium* at seven days of life. These differences were also observed at a later age, even at 6 and 12 months [49], with a higher impact of IAP even than that of later administration of oral antibiotics in infants [50,51].

*Bifidobacterium* is a genus belonging to the Actinobacteria phylum and is regarded as a keystone taxon in the gut microbiota early in life, with a strong eco-physiological impact on microbiota composition and function [52,53,54]. Some species can dominate the gut of breast-fed infants [55,56,57]. Convincing evidence has accumulated showing that the presence of bifidobacteria in the gut is associated with health improvement [53,58]. This is particularly true in the case of infants. In the early gut microbiota, bifidobacteria drive the intestinal microbiome development and their removal or failure to colonise may lead to the development of chronic diseases [52]. Several studies have shown lower levels or reduced relative abundances of bifidobacteria on infant populations in different scenarios, such as prematurity, obesity, later sepsis, colics [19,59,60,61,62]. This occurs because the levels and diversity of bifidobacteria depend on different perinatal factors, as was reported in a recent publication [63]. Among them, antibiotics deeply affected the *Bifidobacterium* genus, which could disrupt the crosstalk with the immune system [44]. However, the impact of IAP on gut-specific bifidobacterial populations is still unexplored. 

To date, the studies exploring this field have focused their investigations on using the 16S rRNA gene sequencing approach, quantification of the *Bifidobacterium* genus by q-PCR, or hybridisation-based technique. However, this has provided limited knowledge on the impact of IAP at lower taxonomical levels. To overcome this limitation, developing next-generation techniques, such as the sequencing of the Internally Transcribed Spacer (ITS) within the rRNA locus [64], was useful as marker of *Bifidobacterium* species, as it has been demonstrated in studies focused on the vertical transmission of bifidobacteria [65], the effect of using donor and own-mother’s milk on the premature bifidobacterial community [66] or the impact of several perinatal factors on the establishment of *Bifidobacterium* species in premature and full-term babies [63].

In this context, by using the ITS sequencing technique as a marker of bifidobacterial (sub)species and q-PCR, we aimed at exploring the effect of the IAP on the establishment and development of the bifidobacterial populations in full-term babies during the first three months of life.

## 2. Materials and Methods

### 2.1. Volunteers and Faecal Samples Collection

The study included 43 Caucasian full-term healthy neonates (22 females/21 males) born after an uncomplicated pregnancy at a gestational age ranging between 38 and 41 weeks (mean 39.2) and recruited at the Neonatology Unit of University Central Hospital of Asturias (Oviedo, Spain). Thirty-four neonates were vaginally delivered and nine were born through C-section. Twenty-seven, twenty-six, twenty-three and twenty neonates were exclusively breast-fed at 2, 10, 30 and 90 days of life, respectively. None received antibiotics, pro- or prebiotics during the sampling time considered for the study. Sixteen mothers received antibiotics during the labour period. Seven of them followed a vaginal labour and were administered with an initial dose of five million units of penicillin followed by 2.5 million units every 4 h until delivery (in most cases, the mothers received three or fewer doses) as prophylaxis, due to confirmed or suspected vaginal colonisation by GBS. Whereas the other nine mothers received a single dose of 2 g of intravenous cefazolin, due to C-Section delivery. The rest of the mothers were culture-negative for GBS, and following a vaginal birth, were not exposed to antibiotics (*control group*). None of the mothers received antibiotics during pregnancy or the postnatal period, other than the above mentioned (Table S1).

Fresh faecal samples were collected at 2 (between 24 and 48 h after birth), 10, 30 and 90 days of age in a sterile container and immediately frozen at −20 °C.

The study was approved by the Regional Ethical Committee of Asturias Public Health Service (SESPA) (Ref. 51/18), and an informed written consent was obtained from each infant’s parents.

### 2.2. Faecal DNA Isolation

Faecal samples were homogenised (1:10 *w*/*v*) in a sterile phosphate-buffered-saline (PBS) solution in a LabBlender 400 stomacher (Seward Medical, London, UK) at full speed for 3 min. Then they were centrifuged for 15 min at full speed to the separate cell pellet and supernatant, which were kept at −20° for further analysis. DNA was isolated from faecal pellets following Qiagen manufacturer’s instructions (QIAmp DNA stool kit, Qiagen GmbH, Hilden, Germany), and kept at −20 °C until use for intergenic ribosomal transcriber spacer (ITS) and q-PCR analyses.

### 2.3. Analyses of Faecal Bifidobacterial Populations by ITS Region Profiling

The isolated DNA was used as a target to amplify and further sequencing the ITS region (the 16S–23S internal transcriber spacer of the ribosomal DNA) as described elsewhere [64]. Briefly, primers Probio_bif_uni and Probio_bif_rev and Illumina technology were used for bifidobacterial ITS region sequencing. Sequences obtained were assembled, filtered and annotated by following an in-house protocol, and an improved bifidobacterial ITS database [66]. The number of reads and relative abundances were determined for each bifidobacterial species in each sample analysed.

### 2.4. Analysis of the Faecal Bifidobacterial Levels by Specific Quantitative PCR

Absolute levels of the most relevant gut bifidobacterial species, *B. bifidum*,* B. breve*,* B. catenulatum*,* B. dentium, B. longum*,* B. angulatum*,* B. animalis* ssp. *lactis* and *B. adolescentis* were determined by q-PCR using primers and methodology described elsewhere [66]. Standard curves were created with pure cultures of each strain, which were grown overnight in MRS medium (Difco, BectoneDickinson and Company, Le Pont de Claix, France) supplemented with 0.25% L-cysteine (Sigma Chemical Co, St. Louis, MO, USA) under anaerobic conditions. Cultures were plate-counted, and DNA isolation was completed following the same protocol used for faecal samples (QIAmp DNA stool kit, Qiagen GmbH, Hilden, Germany). Samples were analysed in duplicates in at least two independent PCR runs.

### 2.5. Statistical Analysis

Results were analysed using the SPSS software version 26 (SPSS Inc., Chicago, IL, USA) and Calypso software (version 8.84) [67] with sum normalisation (TSS) and cumulative-sum scaling (CSS) to account for the non-normal distribution of taxonomic count data [68]. Multivariate redundancy analysis (RDA) was conducted. Species number and alpha diversity indices (Chao1 and Shannon) were calculated and analysed by t-test. To assess the differences in gut bifidobacterial relative abundance and levels related to the use of intrapartum antibiotics, and to avoid bias derived from delivery mode, an ANCOVA test with delivery mode as covariable was conducted. LefSe test (linear discriminant analysis effect size) was also used to detect species features between groups (LDA scores > 2 and significance of *p* < 0.05 as determined by Wilcoxon’s signed-rank test) [69]. Data were considered statistically significant at *p* < 0.05.

### 2.6. Nucleotide Sequence Accession Numbers

The raw sequences from the samples have been deposited in the National Centre for Biotechnology Information (NCBI)—Short Read Archive (SRA) under the BioProject ID code PRJNA750917.

## 3. Result

### 3.1. Bifidobacterium Populations on Full-Term Infants

*B. longum* ssp. *longum* was the most abundant species detected along the three first months of life, followed by *B. breve*, *B. dentium*, *B. adolescentis*, *B. bifidum* and *B. pseudocatenulatum* (Table S2). Although bifidobacterial ITS profiling revealed 35 different (sub)species in the infant population analysed, at the age of two days, there was a large inter-individual variability with babies harbouring a single species (*B. breve*,* n* = 1) and babies with even 30 different ones (*n* = 1). On average, at this age, the babies harboured 11 different bifidobacterial species, with a range between 9 ± 4 and 11 ± 6 (mean ± SD) (sub)species along with the study. *B. longum* ssp. *longum, B. breve* and *B. adolescentis* were the species with the highest average relative proportions (29.18%, 22.14% and 10.18%, respectively) and highest occurrence: *B. longum* ssp. *longum* was present in 98% of babies, *B. breve* in 95% and *B. adolescentis* in 81% at two days of life. At the age of 10 days, *B. dentium* became the third most abundant species and was present in 69% of babies, following the same trend at one month of age. At the three months’ time, we observed that more than 70% of the sequences were assigned to *B. longum* ssp. *longum, B. breve*, *B. bifidum* and *B. pseudocatenulatum* in descending order (Table S2). Absolute determinations corroborated those observations. *B. longum* and *B. breve* showed the highest counts, along with *B. catenulatum* during the first month of life. At three months of age, the count levels of *B. longum* and *B. breve* were followed by *B. bifidum* and *B. catenulatum*, in this order. *B. dentium* and *B. adolescentis* remained at relatively low levels by q-PCR (Table S2), despite showing high relative abundances by ITS-sequencing at some points.

Other than these general observations, clear differences in the bifidobacterial community composition and levels were observed because of the perinatal antibiotic administration, as is depicted below.

### 3.2. Gut Bifidobacterial Diversity Is Affected by Intrapartum Antibiotics

Neonates from the antibiotic group presented an increased number of coexisting bifidobacterial (sub)species at the different time points (11 ± 5 at 2 days, 11 ± 3 at 10 days, 12 ± 4 at 30 days, 10 ± 5 at 90 days, as average mean ± sd) in contrast to the average number of different species observed in the non-antibiotic control group (10 ± 6 at 2 days, 9 ± 5 at 10 days, 8 ± 3 at 30 days, 9 ± 5 at 90 days, mean ± sd). Those differences also reached statistical significance (*p* < 0.01) at one month of life.

**Figure 1:**
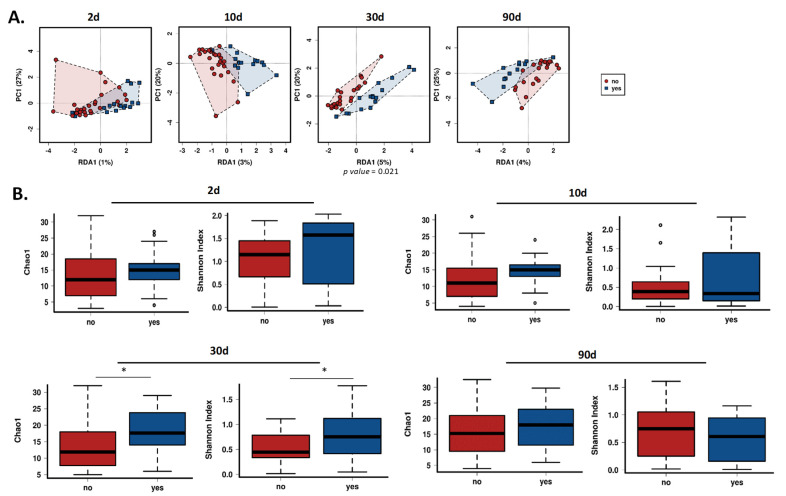
Gut bifidobacterial diversity. (**A**) Redundancy analysis (RDA) and (**B**) Chao1 and Shannon indexes between the control and antibiotic group of babies at 2, 10, 30 and 90 days of life. No: Control group (non-antibiotic); Yes: Antibiotic group. * Indicates statistically significant differences (*p* < 0.05).

With the objective to assess how the intrapartum antibiotic treatment to mothers affects the structure of the gut bifidobacterial community, a multivariate redundant discriminant analysis (RDA) and two indices of alpha-diversity metrics were performed. Statistically significant differences were neither observed during the first days of life, nor at three months (Figure 1). However, at one month of life, both infants’ groups differed between them (RDA *p* < 0.05) (Figure 1A). Moreover, Chao1 and Shannon indices showed higher bifidobacterial alpha diversity in the group of babies whose mothers received antibiotics (*p* < 0.05) (Figure 1B).

### 3.3. Antibiotics Impact on the Bifidobacterium Species Establishment

It was recently described [63] that the delivery mode affects the bifidobacterial establishment. To investigate the impact of the mother’s antibiotic administration on the gut bifidobacterial composition independently of the mode of labour, we used an ANVOCA method controlling the delivery mode as covariable.

**Figure 2:**
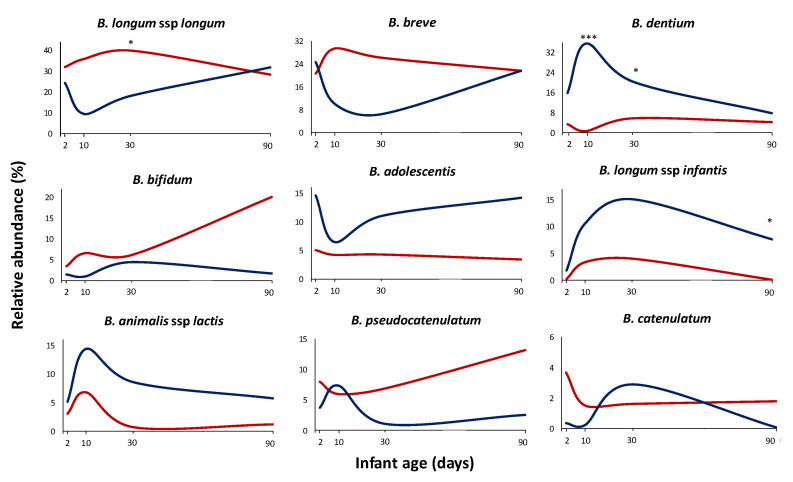
Bifidobacterial relative abundance. Average relative proportions of the dominant (sub)species during the first three months of life in the control group (red line) and antibiotic group (blue line). * Indicates statistically significant differences (* *p* < 0.05; *** *p* < 0.001) at the corresponding sampling times (2, 10, 30, or 90 days of age).

**Figure 3:**
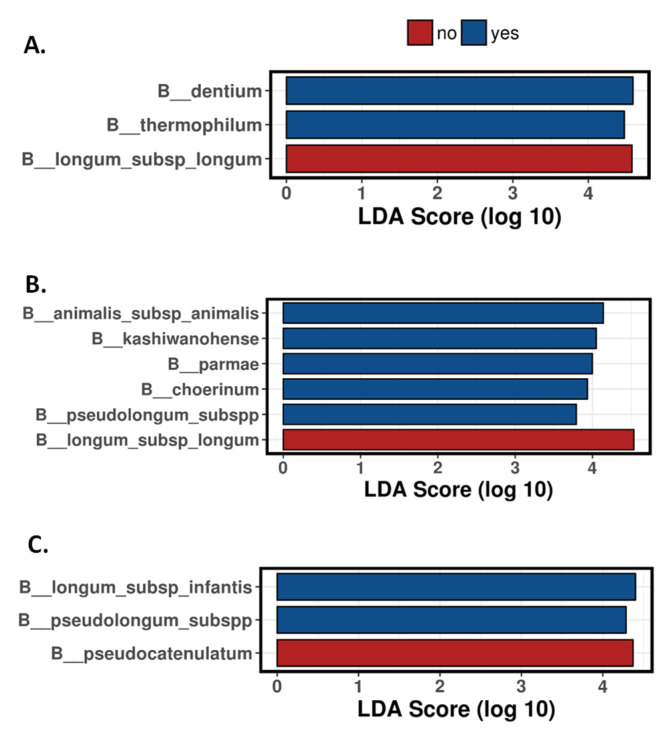
Bifidobacterial composition. Linear discriminant analysis (LDA) scores of bifidobacterial species with differentially abundance between antibiotic and control groups at 10 (**A**), 30 (**B**) and 90 (**C**) days of age. No: Control group (non-antibiotic); Yes: Antibiotic group.

We could observe that the antibiotic administration to mothers in the perinatal time had an impact, at both qualitative and quantitative levels, on developing the bifidobacterial population in the neonate during the first months of life. Overall, the relative and absolute levels of the most abundant species, such as *B. longum*,* B. breve*,* B. bifidum*, or *B. pseudocatenulatum*, were negatively affected by the treatment, whilst *B. dentium* or *B. adolescentis* were increased, this getting statistical significance at some time points (Figure 2, Figure S1). Moreover, other (sub)species with low relative abundance also showed differences among the groups of babies whose mothers received antibiotics with respect to the control group of babies (no antibiotics administered to mothers) (Table S3). For example, *B. pseudolongum* ssp. *pseudolongum* was significantly increased (*p* < 0.05) in the antibiotic group (1.97% of relative abundance; 44% of occurrence) compared to the control group (0.23% of relative abundance; 30% of occurrence) on the second day of life. As with regard to the majority (sub)species (Figure 2) at 2 days of age, *B. adolescentis* showed increased proportions in antibiotic group infants with respect to the control group (relative abundances of 14.58% vs. 5.05% respectively; *p* = 0.068). At 10 days of age, *B. dentium* was significantly higher (*p* < 0.001) in the antibiotic group with respect to the control group (36% vs. 0.63% relative abundance, respectively) (Figure 2). A linear discriminant analysis effect size (LEfSe) (Figure 3) indicated that this bifidobacterial species was also the most discriminant (4.59 LDA score) between the two groups of babies established based on mother’s antibiotic treatment at ten days of age. We also observed that, although not reaching statistically significant differences (*p* = 0.093), *B. breve* decreased in the group of babies whose mothers received antibiotics (10% of relative average abundance) in comparison with the control group (29.48%) (Figure 2). The former observations were further confirmed when absolute levels of these species were determined by q-PCR (*p* < 0.05 for *B. dentium*; *p* = 0.085 for *B. breve*) (Figure S1).

One month of life was the age where more differences between the two groups of babies were observed. *B. longum* ssp. *longum* showed significantly lower relative proportions (*p* < 0.05) in the IAP group (18% vs. 40% in the control group) (Figure 2), which also was confirmed by a reduction of absolute levels analysed by q-PCR (7.21 vs. 8.16 log_10_ cfu/g; *p* < 0.01) (Figure S1). This species also remained at this age as the species was more discriminant (4.53 LDA score) between the two groups of babies (Figure 3). In the same way, the absolute levels of *B. breve* were reduced in the antibiotic group of babies (5.87 vs. 6.96 log_10_ cfu/g; *p* < 0.05) (Figure S1), which was also reflected in its relative abundances (6.40 vs. 26.25% respectively; *p* = 0.097) (Figure 2). In the faecal samples of one-month old babies, the mother’s antibiotic treatment was found to promote higher presence and relative proportions of other species. Thus, *B. dentium*, with an 87.50% of occurrence in the antibiotic group and 66.67% in the control group, showed a significantly (*p* < 0.05) higher proportion in the antibiotic group (20.39 vs. 5.71%, respectively); *B. animalis* ssp. *animalis* was found to be in the 56.25% of the babies whose mothers received antibiotics (12.50% control group) and showed higher relative proportions in these babies (0.68 vs. 0.03%; *p* < 0.05); *B. kashiwanogense*, present in 56.25% of the antibiotic-babies (20.83% control group), also displayed higher proportions in this group (1.81 vs. 0.04%; *p* < 0.05), as well as *B. mongoliense* (0.13 vs. 0.00% relative abundance; *p* < 0.05), *B. parmae* (0.05 vs. 0.00% relative abundance; *p* < 0.01) and *B. reuteri* (0.04 vs. 0.00% relative abundance; *p* < 0.05) (Table S3).

After three months, some of these alterations seem to be reduced, although slight differences persisted (Figure 2, Table S3). *B. pseudocatenulatum* for the non-antibiotic group and *B. longum* ssp *infantis* for the antibiotic group remained as the most discriminant species between groups (Figure 3). Moreover, while ITS results do not reveal differences in *B. bifidum* between the two groups of babies, probably due to the intrinsic variability among individuals, q-PCR analyses still unveiled significantly lower levels (*p* < 0.05) of this species in antibiotic-treated babies at three months of age (Figure S1).

## 4. Discussion

This study analysed the effect of the IAP on the *Bifidobacterium* (sub)species establishment in full-term babies until three months of age. The ITS sequencing technique, which has been previously employed to assess other perinatal factors, such as gestational age, delivery and feeding mode [63], the effect of donated milk on the premature bifidobacterial community [66] or to study the vertical transmission of bifidobacteria [65], allowed us to thoroughly unveil the effect of the IAP—going a step beyond conventional studies which only used 16S sequencing information at the genus level. This technique can be used for tracking bifidobacterial (sub)species in a more accurate way than the use of other techniques. Moreover, the use of specific q-PCR methods for the most abundant bifidobacterial species allowed us to complete the study at a truly quantitative level. Thus, to the best of our knowledge, this is the first study encompassing qualitative and quantitative information about *Bifidobacterium* (sub)species establishment after the use of one of the most common clinical perinatal practices, the IAP.

The study of the microbiome during the last years laid the foundation for considering it as a vital organ or key factor for maintaining a healthy status. Moreover, it is known that its correct establishment at the beginning of life will entail consequences for later life [14,70]. *Bifidobacterium* is one of the dominant genera in the early microbiota and was recurrently linked to a healthy status [53,58]. Alterations on gut microbiota after IAP treatment were repeatedly observed by several authors. At the family and genus level, this antibiotic exposure was linked to the higher relative abundance of members of *Clostridiaceae*, *Enterococcaceae*, *Campylobacteriaceae* families and lower proportions of *Bacteriodes* and *Bifidobacterium* [45,46,47,48]. Moreover, it was observed that the duration of IAP administration exerts a major persistent negative impact on the bifidobacterial genus after 12 weeks of life [71]. *Bifidobacterium* genus is one of the microbial groups severely and negatively impacted by this prophylactic practice; however, its effect at the species level had not been previously explored.

In general, the dominant bifidobacterial species identified in this work, *B. longum* ssp. *longum*, *B. breve*, *B. dentium*, *B. adolescentis*, are in good agreement with those showed as dominant in other studies [63,72,73]. Our results demonstrated that IAP impacts the bifidobacterial community during the first months of life. A multivariate redundant discriminant analysis showed a clear separation between babies whose mothers were administered IAP with respect to the control group, which was evident and significant at one month of life. Moreover, we also found significantly higher alpha diversity in the bifidobacterial populations of the IAP-babies group at the age of one month. Our results are in contrast with that observed when the global gut microbiota community was studied, and the differences with respect to the IAP practice appear in the early time-points analysed, the microbiota becoming more uniform from the first month of life [45,46]. Our data, although interesting, are not surprising, since an increased bifidobacteria diversity when the scenario is not optimum for the correct gut microbiota establishment was previously reported [63,66]. Despite being an every-changing process, early-life gut microbiome development is characterised by low bacterial diversity, unlike that of adults [74,75], and dominance by certain *Bifidobacterium* species reflects a healthy status. However, our results show that when the correct gut microbiota establishment is affected by antibiotics, IAP in this case, the dominance of a few bifidobacterial (sub)species is altered, and the diversity is increased, favouring a non-natural gut environment. We are starting to understand that unfavourable situations early in life could entail changes in the gut microbiome from the whole structure to even at the species level.

IAP treatment also affects the bifidobacterial community at a compositional level. Although in the first days of life, slight differences could be observed among the two groups of infants, at 10 days, a clear differential pattern was evidenced. Thus, *B. dentium* was increased in the antibiotic group—this species was also discriminant for those babies, while *B. breve* showed lower levels in the antibiotic group. These differences become even larger at one month of age. Moreover, at this time, *B. animalis* spp. *animalis* was also significantly increased in babies whose mothers received antibiotics, whereas *B. longum* spp. *longum* was negatively affected by antibiotics. These results are slightly different to those reported by Aloisio et al., the sole work currently available focusing on IAP influence on bifidobacterial species to date [76]. These authors observed a lower diversity and lower abundance of *B. breve, B. bifidum* and *B. dentium* in babies at seven days of life by using the DGGE technique. These differences among both studies may be mainly due to the different techniques used in each case. However, our data suggest a pattern of *Bifidobacterium* species in concordance with that observed by other authors in other undesirable or unideal circumstances. Saturio et al. found lower relative proportions of *B. longum* spp. *longum* and *B. breve* in C-section babies and lower proportions of the former species in preterm neonates [63]. Nagpal et al., using a q-PCR method, also observed lower levels of *B. longum* in C-Section infants [77]. Moreover, when the gut bifidobacterial population were compared between premature babies fed with their own mother’s milk or donated milk, *B. dentium* appears to be significantly higher in babies fed with donated milk [66]. *B. longum* is the species that predominantly inhabit the human intestines [78]. It was shown in our study, and in concordance with other previously published, that when unideal perinatal conditions exist, this species is affected, and its abundance and levels are decreased. On the other hand, it seems that *B. dentium* has an advantage in those negative circumstances and increases its presence. At three months of age, despite the larger differences that appear to be recovered, specific species remain as discriminant signatures in each group of babies. It discloses that the effect of the IAP treatment on *Bifidobacterium* species persists over time.

Our results indicate that IAP treatment impacts intestinal bifidobacteria and that, in good agreement with previous studies, the bifidobacterial community is highly sensitive to suffer aberrancies by different factors commonly present early in life.

## 5. Conclusions

In summary, this study is among the first ones assessing the influence of the IAP in developing the bifidobacterial microbiota in the newborn during the first months of life by both qualitative and quantitative techniques. The data reveals the negative effect of the IAP in some of the most important communities in the early microbiota, such as bifidobacteria. Our results underline specific targets for developing gut microbiota-based products for favouring proper healthy microbial infant development when this clinical practice is required.

## Data Availability

The raw sequences reported in this article were deposited in the NCBI-SRA under the accession number PRJNA750917. Other additional data presented in this study is available upon reasonable request from the corresponding author.

## Supplementary Materials

The following are available online at https://www.mdpi.com/article/10.3390/microorganisms9091867/s1, Figure S1: Bifidobacterial levels. Average levels (log_10_ cfu/g) of the dominant species during the first three months of life in the control group (red line) and antibiotic group (blue line). * Indicates statistically significant differences (** p < 0.05, ** p <* 0.01) at the corresponding sampling times (2, 10, 30, or 90 days of age). Table S1: Basal characteristics of the infant groups included in this study. Table S2: Relative abundance (%) and levels (log_10_ cfu/g) of bifidobacterial (sub)species during the first three months of life in the general population. Table S3: Bifidobacterial relative abundance. Average relative proportions of the minority (sub)species during the first three months of life.
