# Supplementary material for: Effect of Intrapartum Antibiotics Prophylaxis on the Bifidobacterial Establishment within the Neonatal Gut
Source: Microorganisms. 2021 Sep 2;9(9):1867. doi: 10.3390/microorganisms9091867 (PMC8471514; doi:10.3390/microorganisms9091867)
Supplement: Supplementary file 1 [file microorganisms-09-01867-s001.zip › microorganisms-1349451-supplementary/microorganisms-1349451. SUPPL. Figures.pptx]

## Slide 1
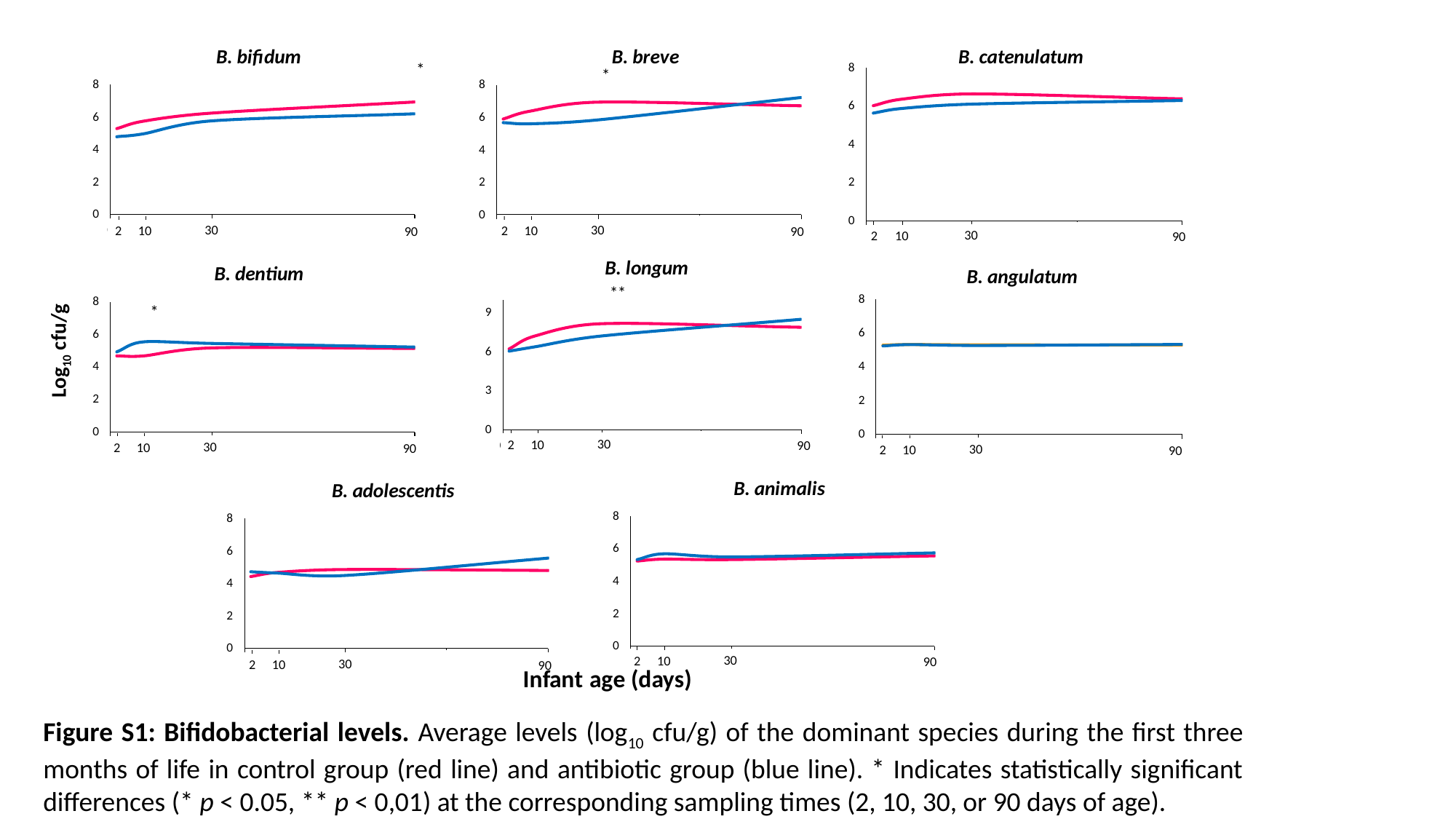

Log10 cfu/g
Figure S1: Bifidobacterial levels. Average levels (log10 cfu/g) of the dominant species during the first three months of life in control group (red line) and antibiotic group (blue line). * Indicates statistically significant differences (* p < 0.05, ** p < 0,01) at the corresponding sampling times (2, 10, 30, or 90 days of age).
